# Retrospective comparative analysis of choledochoscopic bile duct exploration versus ERCP for bile duct stones

**DOI:** 10.1038/s41598-020-71731-2

**Published:** 2020-09-07

**Authors:** Y. Al-Habbal, I. Reid, T. Tiang, N. Houli, B. Lai, T. McQuillan, D. Bird, T. Yong

**Affiliations:** Department of HPB Surgery, The Northern Hospital, Epping, VIC 3076 Australia

**Keywords:** Choledocholithiasis, Cholecystitis, Cholelithiasis

## Abstract

Debate still exists for the management of choledocholithiasis. The purpose of this study is to quantify the rate of recurrent choledocholithiasis post choledochoscopic bile duct exploration (CBDE) in comparison to ERCP and sphincterotomy, and to demonstrate the feasibility of this approach in a busy metropolitan hospital. Data of patients undergoing CBDE from 2009–2014 at the Northern Hospital, Victoria, Australia, was collected retrospectively. Primary outcomes were bile duct clearance rate and rate of recurrent stones post-clearance. Secondary outcomes measured were post-operative complications, laparoscopic to open conversion rate and operative time. Data of patients undergoing ERCP at the same institution was collected and compared. In total, there were 4,091 cholecystectomy cases performed from 2009–2014, of which 260 (6.3%) of patients had an intraoperative cholangiography (IOC) indicating a common bile duct (CBD) stone. Two hundred and forty-eight patients (95.3%) had a CBDE. The remaining 12 patients (4.6%) had radiological clearance, which were excluded from the study. The overall clearance rate for patients undergoing CBDE was 84% (209/248). The risk of recurrent stones up to 8 years post clearance was 2% (4/209). In the same institution, and between 1998–2012, a total of 1,148 patients underwent ERCP, of which 571 had endoscopic sphincterotomy (ES). Forty-three patients required a repeat ERCP for recurrent CBD stones with a complication rate of 7.5%. Time to recurrence ranged from 6 months to 10 years with a mean of 4.5 years. The rate of recurrence was lower in the CBDE group compared to the patients who had an ERCP (8.9% vs. 2%). CBDE is a feasible and effective method for clearance of CBD stones at the time of laparoscopic cholecystectomy. This approach, although not widely used, reduces the need for ERCP, which has inherent complications. In the longer term, this series showed a significant reduction in the rate of CBD stone recurrence.

## Introduction

Debate still exists as to the management of secondary choledocholithiasis. Gallstone-related disease represents a large proportion of elective and emergency work of most general surgical units in Australia. Therefore, efficient and effective treatment of this condition would have a significant bearing on the general surgical workload as well as health care economics. Currently ERCP is the mainstay of treatment of secondary choledocholithiasis. Secondary choledocholithiasis being the migration of gallstones from the gallbladder into the common bile duct. Published reports have shown that there is a subset of patients that re-present with primary choledocholithiasis with a rate as high as 14%^1^, a situation where bile duct stones form primarily in the common bile duct. The morbidity from recurrent primary choledocholithiasis can be significant with repeated bouts of cholangitis, scarring and fibrosis of the tissues around the bile duct and the eventual need for biliary bypass procedures.

The Hepato-Pancreato-Biliary (HPB) Unit at the Northern Hospital, Victoria, Australia had adopted a strategy to treat CBD stones laparoscopically in the first instance. This has been borne out from the findings of the ERCP data published from the same institution with a recurrent CBD stone rate of 8.9%^2^. The postulation is that there is benefit from preserving the sphincter of Oddi as biliary reflux and bacterial contamination of the biliary tree forms a nidus for CBD stone re-formation. This has been demonstrated in several studies^3^. Our paper outlines the data from the cohort of patients who had choledochoscopic bile duct exploration (CBDE), at time of laparoscopic cholecystectomy, for choledocholithiasis at the Northern Hospital. Results were compared to a historical cohort of ERCP patients in the same institution.

## Methods

This is a retrospective, single-centre, observational study. All patients who had CBDE from 2009 to 2014 at the Northern Hospital, Victoria, Australia, were included. This study was approved by the Northern Health Human Research and Ethics Committee. Data collection and analysis were done in accordance with the local guidelines at The Northern Hospital. Patients consents were deemed unnecessary by the research and ethics committee given the nature of the study (retrospective analysis “audit” of de-identified patients).

The primary population for analysis was gathered from a prospectively maintained electronic patient registry. The initial search was conducted using the Medicare Benefits Scheme (MBS) of Medicare Australia, item number for cholecystectomy (30,445). Further information was then obtained from scanned patient files accessible via Clinical Patient Folder Software (Infomedix^©^, Melbourne, Australia). Examination of patient operation reports from their index admission for cholecystectomy was performed to identify patients who underwent CBDE. Discharge summaries were studied to highlight complications associated with CBDE and all subsequent readmissions were examined for secondary complications or stone recurrence.

Data was gathered by three clinicians (YA, TT, IR). Primary outcomes studied were stone clearance rate and stone recurrence rate. Secondary outcomes analysed were complications, post procedure ERCP, and length of procedure. For the CBDE group, this study has used a cut off of 6 months post cholecystectomy to define primary choledocholithiasis^4^. The data on ERCP was collected from a previously published paper^2^ where retrospective data was collected from a prospectively maintained ERCP database from patients who had the procedure between 1998 and 2012. Common bile duct stone recurrence was defined as documented imaging of bile duct stones 6 months after the index admission and clearance^4^, with follow up period up to 31 December 2017 for both these cohorts.

### Operative technique of laparoscopic cholecystectomy and choledochoscopic bile duct exploration (CBDE)

Instruments for CBDE used included the Olympus^®^ 3 mm or 5 mm choledochoscope (Olympus, Tokyo, Japan) and Nitinol^®^ stone retrieval basket (1.5F, 1.7F, and 1.9F) (Boston Scientific, Boston, USA). All cases of CBDE were performed by consultant surgeons (DB, TM, TY, BL, NH) or by trainees under direct supervision of the operating surgeon.

**Figure 1:**
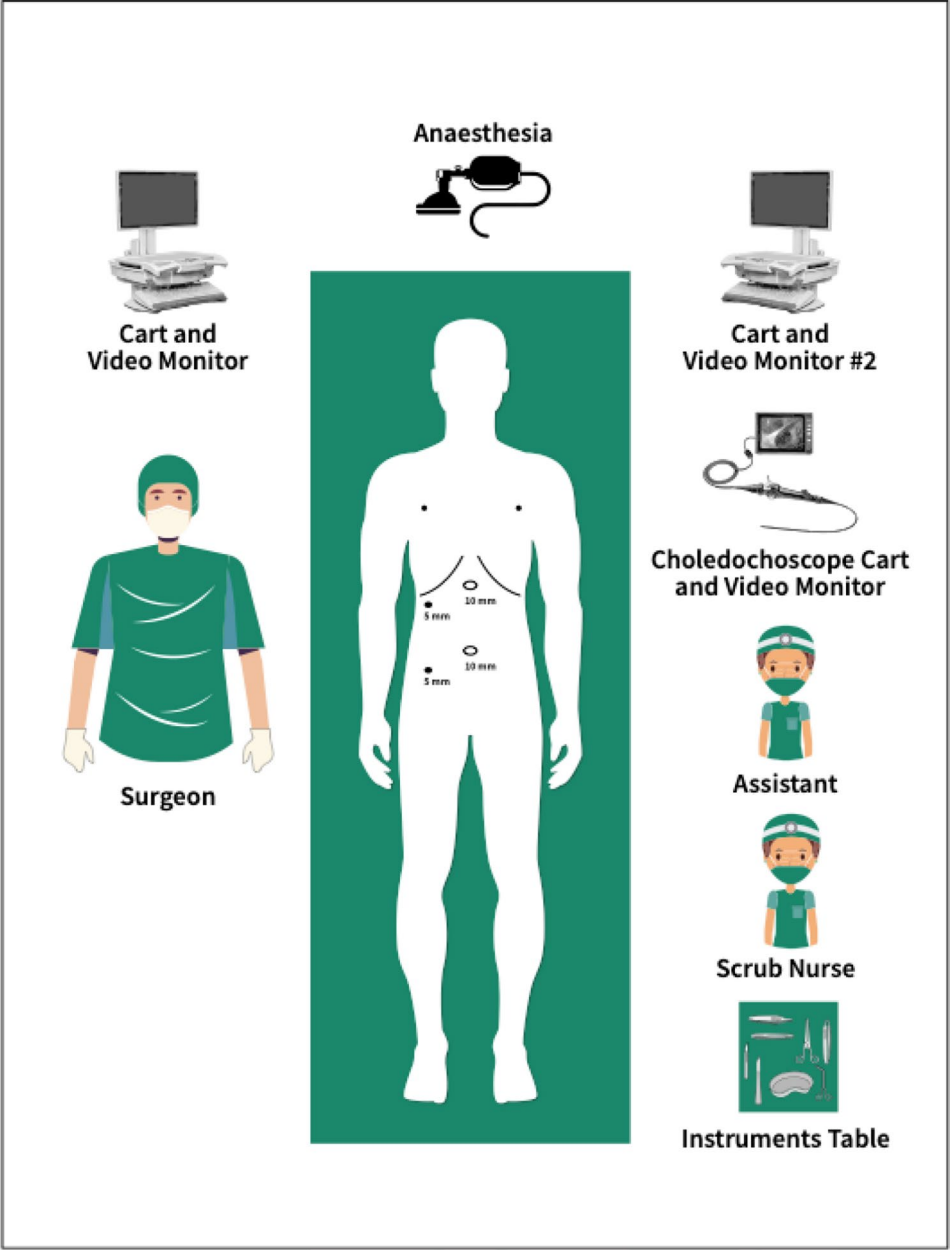
Theatre setup.

The operation commenced as per a routine laparoscopic cholecystectomy. The cystic duct and cystic artery are dissected in Calot’s triangle to obtain the critical view of Strasbourg. A cystic duct opening is then made with a laparoscopic scissors for cannulation with a catheter for cholangiography. Once choledocholithiasis is confirmed, the operating room is set up as per (Fig. 1). The diameter of the cystic duct is then assessed at this point and a decision is made if the 3 mm or 5 mm choledochoscope is utilized for cannulation.

Cannulation of the cystic duct stump with the choledochoscope is performed through either an extra 5 mm port just under the right subcostal margin or with one of the existing working ports. Once in the common bile duct (CBD), the choledochoscope will be pushed distally towards the ampulla. If this step is difficult, then a Jagwire (Boston Scientific) is passed first through a cholangiogram catheter to facilitate passage of choledochoscope as a (Seldinger) technique. Once the stone(s) identified, it will be captured and removed using a Nitinol 1.5F, 1.7F or 1.9F basket, passed down through the working channel of the choledochoscope.

**Figure 2:**
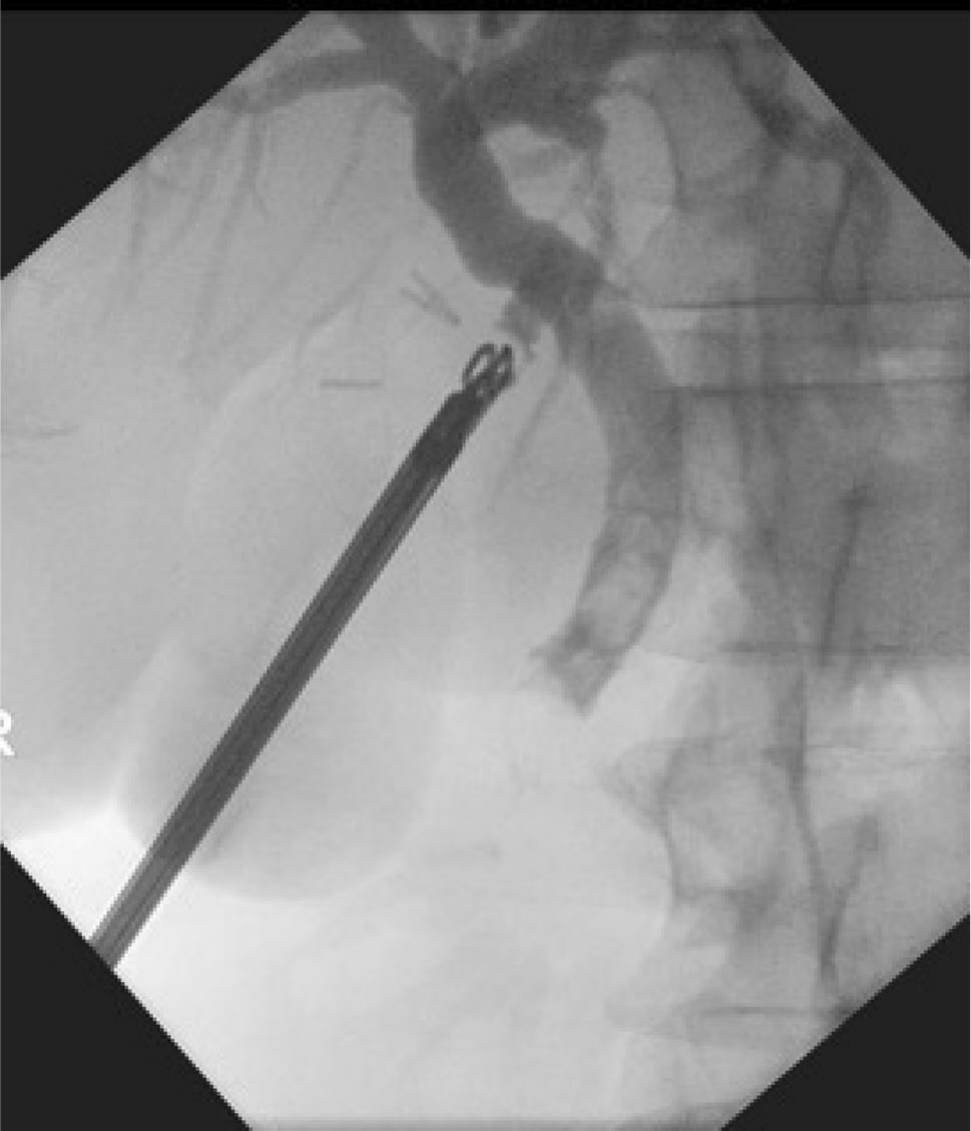
Pre-exploration cholangiogram.

**Figure 3:**
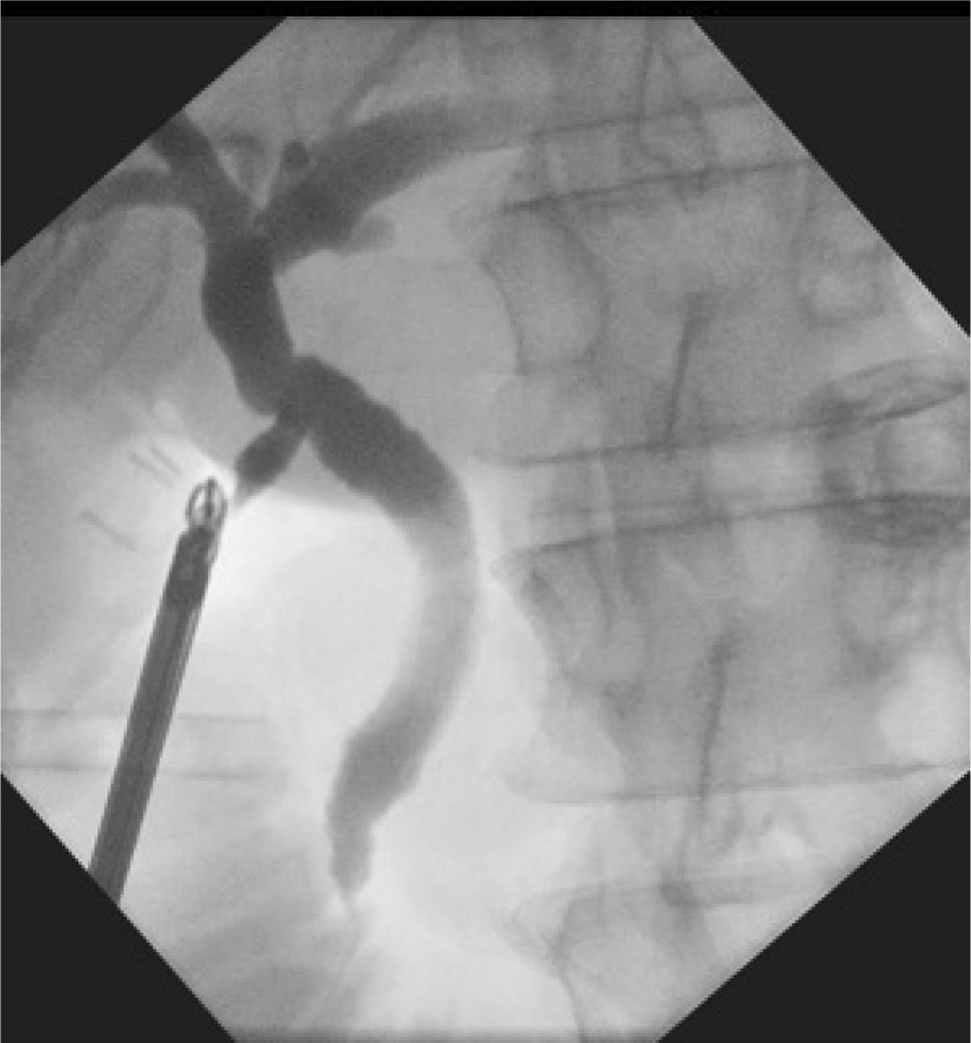
Post-exploration cholangiogram.

Check choledochoscopy upstream and downstream from site of cannulation of CBD was performed at the end of the exploration to rule out the presence of retained stones. If check choledochoscopy was not feasible, especially upstream, then an operative cholangiogram was performed to verify duct clearance. The use of internal biliary stent and post-operative drain tube were utilized at the discretion of the surgeon. An example of trans-cystic pre-exploration cholangiogram (Fig. 2) and post exploration cholangiogram (Fig. 3) is shown below. In a minority of cases, Nathanson’s trans-cystic bile duct basket (Cook Medical, Bloomington, Indiana, United States) was employed. These were excluded from the study.

There were cases that required a choledochotomy for CBD clearance, mainly due to an inability to cannulate cystic duct, or due to presence of large bile duct stone(s). Ideally, the CBD has to be > 8 mm in diameter to be able to progress to choledochotomy. Port positions were similar as described above. CBD was dissected to expose the supra-duodenal portion for about 2–3 cm. A convenient spot on the anterior surface of the CBD was chosen for a longitudinal choledochotomy made with a pair of laparoscopic scissors. The choledochotomy is usually made to the size of the largest stone. Choledochoscope would be inserted via this choledochotomy. Stones were extracted using similar baskets as mentioned before, passed down the channel of the choledochoscope. Once duct clearance was confirmed with check choledochoscopy, the choledochotomy was closed with interrupted 4/0 monofilament non-absorbable sutures. The use of T-tube, internal biliary stent or peritoneal drain was at the discretion of the operating surgeon.

### Comparative analysis

To perform a comparative analysis historical data from a prior study was utilised. The ERCP and sphincterotomy cohort has been derived from the data collected from Nzenza et al.^2^. In that study done at the same institution, a total of 1,148 patients underwent ERCP between 1998–2012, of which 571 had endoscopic sphincterotomy (ES). Forty-three patients required a repeat ERCP for recurrent CBD stones with a complication rate of 7.5%. Time to recurrence ranged from 6 months to 10 years with a mean of 4.5 years. A table outlining the summary statistics of the comparative analysis for the two cohorts is displayed below (Table 1). To perform a comparative analysis, a multivariate logistic regression model was applied to determine if method of stone clearance has a significant effect on the rate of recurrent CBD stone formation. The model employed adjusts for the heterogeneity in the population as often seen in retrospective analysis. Importantly this model is able to factor follow up time interval into the regression model.

$${\ln}\left( {\frac{P}{{\left( {1 - P} \right)}}} \right) \approx \beta_{0} + \beta_{1} X_{1} + { }\beta_{2} X_{2} + \beta_{3} X_{3} + \beta_{4} X_{4}$$

**Table 1:** Summary of the multivariate logistic regression analysis.

	Estimate	Std. error	z value	p-value
(Intercept)	− 17.330	1,027.461	− 0.017	0.987
Age	− 0.003	0.010	− 0.305	0.761
Gender F	13.247	1,027.461	0.013	0.990
Gender M	13.881	1,027.461	0.014	0.989
ERCP vs CBDE	2.374	0.766	3.101	0.002
Follow up time	− 0.051	0.070	− 0.729	0.466

P being the probability of recurrent CBD stone formation, β_1_: age, β_2_: sex, β_3_: follow up time (months), β_4_-Stone clearance method (ERCP versus laparoscopic cholecystectomy and CBDE).

## Results

### Study population

Four thousand and ninety-one patients were admitted to the Northern Hospital for a cholecystectomy between 2009 and 2014. Mean age was 48 years old (14–98 years old). Gender distribution was female:male 70:30.

Routine cholangiogram (IOC) is performed at the time of laparoscopic cholecystectomy at this institution. Out of 4,091 patients, 260 (6.3%) had an IOC indicating bile duct stones. Two hundred and forty-eight patients (5.9%) proceeded to a CBDE, with pre-operative diagnosis listed in (Table 2). Twelve patients had their stones retrieved using radiological methods (Nathanson’s basket or Fogarty balloon). These were excluded from the study.

**Table 2:** Pre-operative diagnosis.

Diagnosis	Number [percentage]
Cholangitis	2 [1]
Cholecystitis	39 [15]
Choledocholithiasis	201 [77]
Gallstone pancreatitis	13 [5]
Mirizzi	5 [2]
Total	260

**Figure 4:**
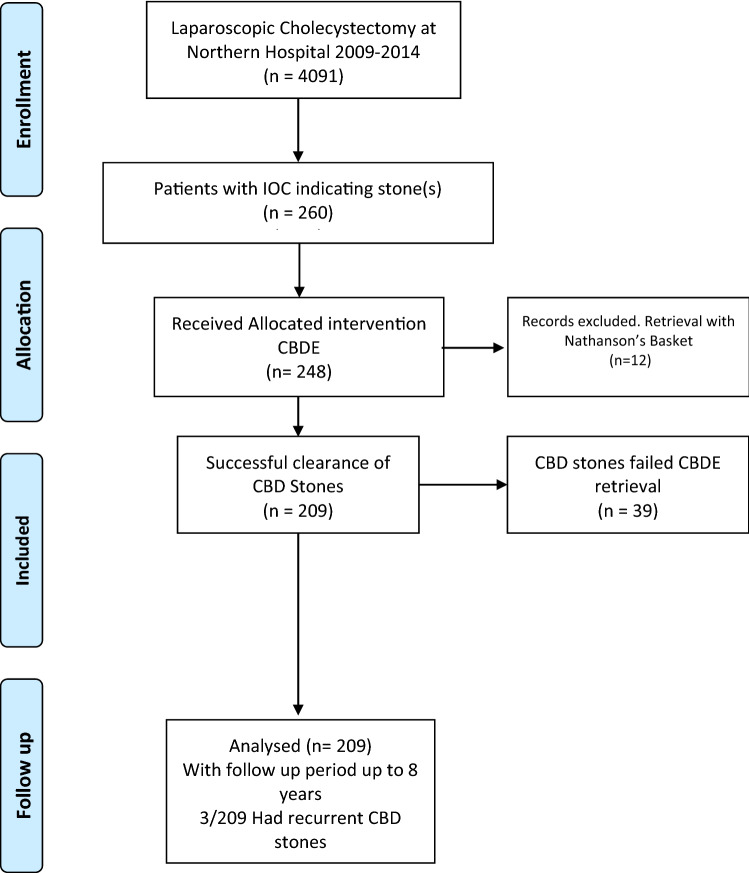
PRISMA flow chart for cohort study design.

The PRISMA chart summarises the enrolment criteria, allocation, follow up and results analysis (Fig. 4).

### Operative outcomes

Of the 248 patients that proceeded to CBDE, 209 had stones successfully cleared via a laparoscopic choledochoscopy giving a clearance rate of 84% (209/248). The risk of recurrence of CBD stones noted over these 8 years was 1.4% (3/209).

The vast majority of these explorations ended with successful laparoscopic trans-cystic stone(s) retrieval. Some cases had to be converted to open, or had choledochotomy instead of trans-cystic retrieval (Table 3).

**Table 3:** Peri-operative outcomes.

	Number [percentage]
Laparoscopic converted to open	24 [9]
Choledochotomy	38 [15]

The most common reason for failed laparoscopic clearance of CBD stones was inability to cannulate the cystic duct. The other causes for failed clearance are outlined in (Table 4).

**Table 4:** Reasons for failed laparoscopic clearance of CBD.

	Number [percentage]
Unable to cannulate cystic duct	9 [23]
Abnormal taper of CBD	5 [13]
Failed basket retrieval of stone	3 [7.7]
Impacted stone at ampulla	3 [7.7]
Avulsed cystic duct	1 [2.6]
Not attempted	4 [10]
Bleeding	2 [5]
Equipment not available	3 [7.7]

Operative time (for cholecystectomy and bile duct exploration) ranged from 88 to 525 min, with the mean time being 180 ± 75 min. The reasoning for operative time fluctuations can be attributed to the experience of each surgeon in CBDE. As the roster increases and more surgeons become proficient in this technique operative times do reduce as shown in the table below (Table 5).

**Table 5:** Effect of surgeon cumulative experience on clearance rates.

Year	Cleared	Not cleared	Total	Clearance (%)	Cumulative clearance (%)	Mean operating time (min)
2008	2	1	3	67	67	182
2009	40	1	41	98	98	201
2010	19	8	27	70	87	188
2011	38	7	45	84	86	170
2012	34	5	39	87	86	186
2013	42	11	53	79	84	175
2014	62	21	83	75	82	160

The complication rate from CBDE in this cohort was 2.8% (7/248).

Table 6 described post-operative morbidity from this cohort. No patient was re-admitted for retained stones within 6 months after a successful CBDE clearance. Four patients experienced bile leak, 3 of which had a choledochotomy performed. One patient had a bile leak progressed onto ERCP to control the leak. A second patient was taken back to theatre where an area of necrosis at the level of the cystic duct clips was found. The cystic duct was further dissected and another clip placed across it below the leak site. The 2 other patients with bile leaks were successfully managed conservatively. Two patients had postoperative bleeding with one requiring conversion to an open procedure to stop a bleed from a posterior sectoral branch of the right hepatic artery. The second patient had bleeding from the gallbladder fossa requiring a re-look laparoscopy and washout.

**Table 6:** Morbidity of CBDE.

	Number [percentage]
Bleeding	2 [0.8]
Bile leak	4 [1.6]
Intra-abdominal collection	1 [0.4]

Twenty-four patients had a laparoscopic converted to open bile duct exploration (9%) with the indications outlined in Table 7. The availability of the 3 mm choledochoscope was paramount with two of the cases requiring conversion to an open approach, not having a 3 mm choledochoscope available and a cystic duct unable to be cannulated with a 5 mm choledochoscope. The other indications ranged from bleeding to 4-quadrant purulent peritonitis with one other patient requiring conversion to close the laparoscopic choledochotomy.

**Table 7:** Indications to convert to open procedure.

	Number [percentage]
Impacted stone in distal CBD	9 [37.5]
Unable to cannulate cystic duct	5 [21]
Intra-abdominal adhesions	3 [12.5]
Other	7 [29]

### Comparative outcomes between ERCP and CBDE

Comparing the 2 methods of stone clearance (ERCP vs CBDE) there is a statistically significant effect on probability of recurrent CBD stone formation after clearance. This result is significant after adjusting for Age, Sex and Follow up time interval with a p-value of 0.002. The Odds ratio has been calculated from the estimates of the logistic regression analysis. The Odds ratio of 10.74 indicates that recurrent CBD stones are 10.74 times more likely when cleared via ERCP and sphincterotomy than trans-cystic exploration in this study, adjusting for Age, Sex and most importantly follow-up time interval. The 95% confidence interval for this estimate is 2.4–48.2.

## Discussion

Gall bladder and bile duct stones are amongst the most common surgical presentations. CBD stones can be predicted in the pre-operative workup. Different scoring systems have been used to predict presence of CBD stones. They are sensitive, but not specific. Bilirubin level, bile duct diameter and alkaline phosphatase (ALP) level were the three independent predictors of CBD stones^5^. Imaging modalities such as ultrasound (US), computed tomography (CT), magnetic resonance cholangiopancreatography (MRCP) and endoscopic ultrasound (EUS) are often used to detect the presence of CBD stones with varying sensitivity and specificity.

The gold standard procedure to treat CBD stones is ERCP. It is still the most commonly performed procedure in Australia to deal with CBD stones in the setting of an intact gallbladder^6^. However, it is associated with short and long-term complications, especially if done with a sphincterotomy. Recurrent stone rates have been quoted at 10–15% in the literature^7–9^. Balloon dilatation of the sphincter carries similar risks^10^. Balloon dilatation alone could also be associated with recurrent stone disease of 13.5% over a 10-year follow up period^11^.

There is an increasing evidence to manage CBD stones laparoscopically, especially in absence of cholangitis^12^. The first successful series of bile duct exploration was described by Phillips et al.^13^. Bile duct stones clearance rate by laparoscopic approach was reported to be as high as 90% in a retrospective study by Martin et al.^14,15^ which compared favourably with this study which showed a clearance rate of 84%. Complications after CBDE documented in the literature is in the range of 7%. This included intraoperative and post-operative complications. Intraoperative complications included jammed basket and all other complications of laparoscopic cholecystectomy. Post-operative complications included bile leak, bleeding and intra-abdominal collections. The complication rate in our cohort was 2.8%, which again compares favourably with the published literature.

With the new advents in digital imaging technology, design of small sized cameras is now feasible and affordable. This has contributed to improved visualization and extraction of bile duct stones^16^. Seeing bile duct stones and removing them under direct vision has the advantage of having visual evidence of clearance rather than using radiological imaging which relies on a column of contrast to indicate no filling defect. The false negative rate of intraoperative cholangiography ranges from 1.4 to 5.4% in the literature^1^.

Bile duct stone recurrence was noted in 4 (2%) patients in this cohort, which contrasted sharply with bile duct stones recurrence rate reported post ERCP. Stone recurrence post-ERCP could be as high as 25%^17,18^. In the author’s centre, the reported CBD stone recurrence after ERCP is 8.9% which is significantly higher in comparison^2^. The possible explanation of this difference could be explained by the fact that bile duct exploration preserves the peri-ampullary sphincter and this in turn maintains the micro-environment of the distal CBD, avoiding reflux of enteric contents and crystallisation of bile^19^.

One of the limiting factors for trans-cystic bile duct exploration is discrepancy between the stone size and the calibre of the cystic duct. In this situation, trans-ductal approach is indicated. In this series, 38 patients (15%) had a choledochotomy for stone retrieval. Three of the 38 patients had bile leak (7.8%). Trans-ductal approach is well documented in the literature with good success rate of stone clearance^20,21^.

Clearing the bile duct at the same time of doing laparoscopic cholecystectomy is recommended for in patients with gall stones and bile duct stones. In addition to the advantage of preserving the periampullary sphinctre, this approach has the advantage of avoiding exposing the patient to multiple procedures and anaesthetics. If CBDE fails, one could always default back to clearing the duct with ERCP.

To the authors knowledge, this is the largest study in choledochoscopic bile duct exploration in the literature and with the longest follow up period. However, the main limitation of this study is that it is a retrospective study. Follow up of patients could be inaccurate as there are other hospitals in reasonably close proximity to our centre that provides ERCP services as well. Therefore, patient drift may be a factor in not capturing patients with recurrent stones.

## Conclusions

There appears to be benefit in preserving the peri-ampullary sphincter based on results from this study. There is a significantly reduced rate of recurrent CBD stones post CBDE compared to ERCP. Furthermore, the results here showed that laparoscopic cholecystectomy and CBDE, and with adequate training and exposure, is a viable and feasible approach for choledocholithiasis. The authors would recommend that CBDE be performed to protect them from recurrent stones post ERCP.
